# Effects of High-Frequency Proprioceptive Training on Single Stance Stability in Older Adults: Implications for Fall Prevention

**DOI:** 10.1155/2019/2382747

**Published:** 2019-05-22

**Authors:** Dario Riva, Mara Fani, Maria Grazia Benedetti, Angelo Scarsini, Flavio Rocca, Carlo Mamo

**Affiliations:** ^1^International Society of Proprioception and Posture, Torino, Italy; ^2^Proprioception Center, via Valgioie 85-87, 10146, Torino, Italy; ^3^South-East District, Local Health Unit Città di Torino, Torino, Italy; ^4^Physical Medicine and Rehabilitation Unit, IRCCS-Istituto Ortopedico Rizzoli, Bologna, Italy; ^5^Tolmezzo Hospital, Local Health Unit AAS3, Tolmezzo (UD), Italy; ^6^Epidemiology Unit, Local Health Unit TO3, Piemonte Region, Grugliasco, Italy

## Abstract

Single-limb stance instability is a major risk factor for falls in older adults. Thus, improvement of stance stability could play an important role in fall prevention. This study aimed to determine whether high-frequency proprioceptive training (HPT) could significantly improve single stance stability (SSS) in older adults, by increasing proprioceptive control and optimizing the contribution of vision. Sixty-one subjects (30 men, 31 women) aged 65-85 years were investigated. The subjects were randomly assigned to three intervention groups, i.e., HPT, treadmill, and no intervention, stratifying by gender and proprioceptive control at baseline. Stability tests and HPT, consisting of 12 sessions (6 weeks), were performed with computerized postural stations. Pre-post analysis showed that HPT significantly improved SSS by increasing proprioceptive control (p<0.001) and postural control (p<0.01). The treadmill and no intervention groups did not show any significant change. The results showed that different levels of proprioceptive control may activate, inhibit, or minimize the stabilizing intervention of vision. Given that HPT significantly reduced ankle sprains and low back pain in professional athletes (previous study), we discuss the hypothesis that the risk of falls in older adults and the risk of recurrent injuries in athletes would have a common origin: lack of proprioceptive control consequent to reduced interaction with uneven ground. The findings suggest that HPT may be a powerful activator of refined proprioceptive control, which allows increased SSS, safer interaction with the ground, and mitigation of other risk factors.

## 1. Introduction

In developed countries, falls are the leading cause of injury in adults over the age of 65 years [1]. The risk of falling increases with age because of extrinsic and intrinsic reasons. Among the intrinsic factors, single stance instability is a major risk factor for falls and loss of independence [2, 3]. Older adults show important changes in spatial and temporal gait parameters: decreased stride length and speed, decreased single support time, and increased stride width [4–8]. Some authors consider these changes consequent to aging [8–10], whereas others consider them as stabilizing adaptations to fear of falling and instability [5, 8, 9, 11, 12]. Nevertheless, these gait changes have been shown to be risk factors for falls in prospective studies [6, 12]. The fact that the single-limb support period accounts for 80 % of the gait cycle at normal walking speed [13, 14] highlights the importance of single stance stability to guarantee the safety of basic movements, such as walking or going up and down the stairs. Single stance stability depends on the effectiveness of the stabilizing muscles and primarily on the strength of the extrinsic and intrinsic muscles of the foot. Moving on uneven surfaces is the natural way to activate the reflex contractions of the stabilizing muscles and to make them stronger. Age-related decline in single stance stability would be a consequence not only of aging but even more of proprioceptive disengagement [15, 16], which is due to the hyper-use of vision as stabilizer and to the growing lack of interaction with uneven ground [17]. Moreover, the anatomical and functional complexity of the visual system exposes it to aging [18], with a decline in visual acuity, poor contrast sensitivity, impaired depth perception, and restriction of the visual field. In addition, older people are susceptible to developing visual deficits from common eye pathologies including cataracts, macular degeneration, and glaucoma [19]. On the contrary, the redundancy of the proprioceptive receptors [20] makes proprioceptive control anatomically less sensitive to aging. Hence, reduced interaction with uneven ground and the consequent lack of proprioceptive control would represent the initial trigger in the causal chain that leads to a growing intrinsic risk of falls in older adults. Thus, we hypothesized that a refined and enduring proprioceptive control in single stance would decrease the instability, allow safer interaction with the ground, and mitigate the other risk factors. The purpose of this study was to determine whether quantifiable high-frequency proprioceptive training (HPT) in older adults could significantly improve single stance stability by increasing proprioceptive control and optimizing the contribution of vision.

## 2. Materials and Methods

### 2.1. Subjects

This study recruited male and female residents in Turin (an industrial city in Northwestern Italy) aged 65–85 years between March 2013 and February 2014 using flyers and posters of invitation in the clinic of general practitioners of the Local Health Unit. Subjects were screened via a phone interview based on the following inclusion criteria: subjects should be able to walk for at least 6 m without an assistive device, could perform the tests in this study, and had not performed any proprioceptive or balance training in 2 years prior to the study. Before the tests, a medical history questionnaire was administered to obtain data on the subject's health status. Subjects with cognitive deficits or health problems were excluded only if the condition could compromise their collaborative capacity or is associated with potential rapid impairment. Sixty-one older adults (30 men, 31 women) were included in the study. Each gender group was divided into two strata according to the level of proprioceptive control. The stability index (SI) in eyes closed (EC) condition was the marker of proprioceptive control. The SI is a score (0-100 %) that takes into account instability and precautionary strategy (hand support). The SI is described in the* Procedures* section and was comprehensively explained in a previous article [17]. In our study, the SI range of stratum A was 0–30 % (women, 40 %; men, 30 %), while the SI range of stratum B was 30–70 % (women, 60 %; men, 70 %). The SI upper limit of 70 % was chosen because in a previous study [17], it was noted that men and women with proprioceptive control ≥70 % did not fall. The SI upper limit of 30 % for stratum A was chosen because under this value the low level of proprioceptive control tends to inhibit visual gain. This behavior emerged in a previous study [17] and was then confirmed by our everyday experience. After baseline assessment, the subjects were randomly assigned to three intervention groups: HPT, treadmill, and no intervention groups, stratifying by gender and proprioceptive control at baseline. The characteristics of the study subjects are summarized in Table 1. Both assessors and participants were blinded to the group placement at the time of baseline assessments. Figure SM (Supplementary Material (available here)) shows the flow of the participants throughout the study following baseline testing and randomization. This study was approved by the Local Ethical Committee ASLTO1 and conducted in accordance with the Declaration of Helsinki. All subjects were informed of the benefits and risks of the investigation prior to signing an institutionally approved informed consent document to participate in the study.

### 2.2. Procedures

This study used the method of Riva et al. and the methods description partly reproduces their wording [17, 21].

#### 2.2.1. Instruments

Stability tests and HPT were performed using the Delos Postural Proprioceptive System (DPPS; Delos, Turin, Italy) [22]. Each station, which was connected to a personal computer with specific software (DPPS 6.0), included an electronic rocking board, an electronic postural reader, an infrared sensor bar, and a display. In case of risk of falling, the subject could touch the bar placed in front of him to regain vertical control rapidly. The bar was equipped with an infrared sensor that could detect when the subject touched it for support. The electronic postural reader (Delos Vertical Controller, DVC), which was applied to the sternum, measured trunk inclination in the frontal (x) and sagittal (y) planes by means of a two-dimensional accelerometer unit. The test was performed using the DVC and the station with the sensorized bar. The electronic rocking board (Delos Rocking Board, DRB) had a single degree of freedom on the frontal plane (range of motion: ±15°) and measured the inclination of its moving plate. The mechanical characteristics of the rocking board are summarized in Table 2 [22] and have been described in a previous study [23]. Instability of the rocking board could be decreased by changing the rolling radius (from 55 mm to 80 or 110 mm). This option facilitated the first approach to the proprioceptive station and made the transition to higher levels of instability easier. DRB was used only for training.

#### 2.2.2. Algorithms

Data from the postural reader and the rocking board are a stream of acceleration samples obtained by converting the sensor outputs into the digital domain; at a rate of 100 Hz. Rocking error indicates the average inclination of the board in relation to the horizontal plane. The equations involved in the management of the data from the devices have been described in two previous studies [17, 21]. Moreover, postural assessment was based on the measure of postural instability (PI), which derives from the average instability in the frontal and sagittal planes. PIxy (expressed in degrees) is an indicator of the average amplitude of the postural cone of instability.

#### 2.2.3. Single Stance Stability Assessment

**Figure 1:**
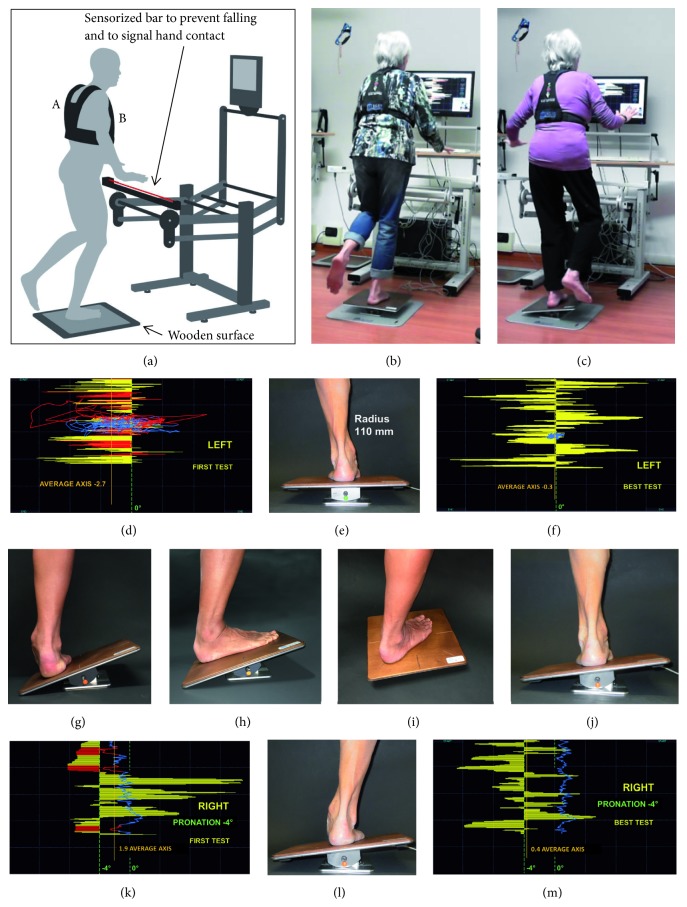
(a) The postural proprioceptive station [17]. The red line represents the infrared ray of the sensorized bar. Vest (A) to support the “postural reader” in (B) sternal position. (b, c) A subject on the electronic rocking base in single stance during a training session. (d) The real-time trace (yellow bars) of the rocking base (e) and the trace of the postural reader (blue line). Note the presence of precautionary strategy (red trace) and hypersupination in the first test (d) vs. the best test (f). (g, h) Static exploration of the ankle range of motion: maintaining vertical stability with the weight-bearing ankle in hyperpronation and in maximum dorsiflexion. (i) Orientation of the rocking base at -45° to affect different ranges of motion. (j, k, l, m) Dynamic exploration of the ankle range of motion: (j) supination (inversion); (k, l, m), attempting to maintain 4° of inclination (pronation, eversion). All subjects were asked to minimize postural instability (blue line).

Single stance stability was assessed with a static single stance test [17]. The static test was performed with eyes open (EO) and closed (EC). The subject was barefoot and was asked to minimize PIxy (amplitude of the postural cone) while staying in single stance on a stable wooden surface (Figure 1(a)). No feedback on postural stability was provided during the test. Each trial lasted 20 s, which was followed by a 15-s rest period. The static single stance test consisted of six trials (two with EO and four with EC), in an alternate sequence of the left and the right limb. The average value of the two limbs for all variables was considered.

The postural reader was metrologically characterized through calibration for comparison with a reference triaxial microelectromechanical system accelerometer used as reference standard. This standard was calibrated at Istituto Nazionale di Ricerca Metrologica, which is the National Metrology Institute (NMI) of Italy, with a procedure that was validated through a comparison with that of other NMIs worldwide. Based on the calibration results, the expanded uncertainty (with a confidence level of 95 %) of the postural reader was 0.18, which was within the operative and calibration ranges [23].

#### 2.2.4. Stability Index

SI is a score (0–100 %) that is based on two components: autonomy and average postural instability (PIxy, cone of instability). SI is capable of ranking all kinds of performances from the highest (SI >90 %; extremely narrow cone of instability and complete autonomy) to the lowest (SI <30 %; extremely low autonomy with cones that become narrower and narrower) level. High SI values in EC trials correspond to refined proprioceptive control, the expression of effective proprioceptive reflexes that stabilize the subject rapidly before activation of vestibular responses [17]. Lower SI values in EC trials may possibly involve the vestibular system; nevertheless, such values, in any case, are the expression of rougher proprioceptive control. As the vestibular system has a higher threshold of intervention and takes longer before becoming active, it can intervene only if proprioceptive control is not refined and, therefore, permits a longer period for activation. A detailed description of SI calculation was presented in a previous study [17].

#### 2.2.5. High-Frequency Proprioceptive Training

HPT consisted of 12 sessions (two sessions a week for 6 weeks, each session lasting 45 min) and was based on the active management of high-frequency rocking instability (rolling + inclination) of the board (Figure 1). The real-time visual trace on the monitor (it is the feedback of the rocking movements of the base and acts as a feedforward) tracks the subject and notably increases the frequency of corrections (inversions) of the rocking platform inclination. The postural reactions change from macro-amplitude at low frequency to micro-amplitude at high frequency (Figures 1(d) and 1(f)]. On the same rocking base, but without visual feedforward, the same subject would experience instability only at low frequencies. The instability of the rocking base and the resulting postural instability of the subject were recorded. The sessions consisted of a sequence of repetitions lasting 30 s, alternating the left and right limbs in a single stance. The recovery period between repetitions was 15 s. The sequential targets of the proprioceptive training were as follows:

- reduced precautionary strategy (hand support on the sensorized bar, Figures 1(d) and 1(f)),
- improved vertical control based on proprioceptive reflexes,
- enhanced visual gain (VG) when the contribution of vision to stability is limited by an extremely low proprioceptive control level,
- optimized postural control by activating more effective proprioceptive reflexes (proprioceptive control) and minimizing the contribution of vision to stability.

The proprioceptive training was based on the following key concepts.

- Ankle mobility improvement: the rocking base is used as an inclined plane to experience maximum pronation and supination, plantarflexion, and dorsiflexion (15°) in a static condition (Figures 1(g) and 1(h)).
- High-frequency instability of the rocking base (the subject's interaction with the visual feedforward increases the frequency of correction of board inclination and rolling, thereby creating many more situations to be managed).
- Feedback of vertical control.
- Assignment of specific tasks concerning board control and postural control. The exercises with the rocking base were performed in various orientations of the support surface to affect different ranges of motion of the ankle (Figure 1(i)).
- Tasks for exploring the ankle range of motion dynamically: the visual information allowed for the assignment of specific tasks, such as maintaining the rocking base at an inclination corresponding to a certain level of pronation or supination, dorsiflexion, or plantarflexion (Figures 1(j), 1(k), 1(l), and 1(m)) or passing from one inclination to another.
- Hyper-frequency instability: more details of the rocking movement were shown by zooming the trace on the display. The greater number of details led the subject to further increase the frequency of correction.

The last three proprioceptive training sessions consisted of a sequence of longer trials lasting 40 s, alternating the left and right limbs. The recovery period between repetitions decreased from 15 to 10 s. The actual training time progressively increased from 18 to ≥22 min. The training density (actual training time/session duration) increased from 40 % to ≥50 %.

#### 2.2.6. Treadmill Walking

The treadmill group performed 12 sessions (two sessions a week for 6 weeks) of walking on a treadmill. The training speed in each session was calculated by increasing the usual speed of the 6-m test by a correction factor (1.8 % at the first session, 3.6 % at the second session, 5.4 % at the third session, and up to 18 % at the tenth session). The purpose was to propose an increasing training speed in each session tailored to the functional level of the subject. All sessions consisted of four walking blocks, with 3-min rest for passive recovery (seated). In each session, all blocks were equal; in each block, the subject started approaching the training speed (1 min), continued maintaining the training speed (3 min), and decreased the speed until completion (1 min). Each session lasted 29 min with 20 min of walking time.

#### 2.2.7. No Intervention

The participants were asked to maintain their usual physical activity and walking time.

### 2.3. Statistical Analysis

Analysis of variance was used to analyze the differences between group means. When comparing the effect of intervention within every intervention group (baseline test value vs. best postintervention test value on the same subject), the paired t-test was used. The normal distribution was verified with the Shapiro-Wilk test. Analyses were stratified by gender and proprioceptive control (SI in EC condition) at baseline. The values of p were two-tailed, and p≤0.05 was considered statistically significant. All analyses were performed using MedCalc Statistical Software version 18.2.1 (MedCalc Software bvba, Ostend, Belgium; http://www.medcalc.org; 2018).

## 3. Results

**Figure 2:**
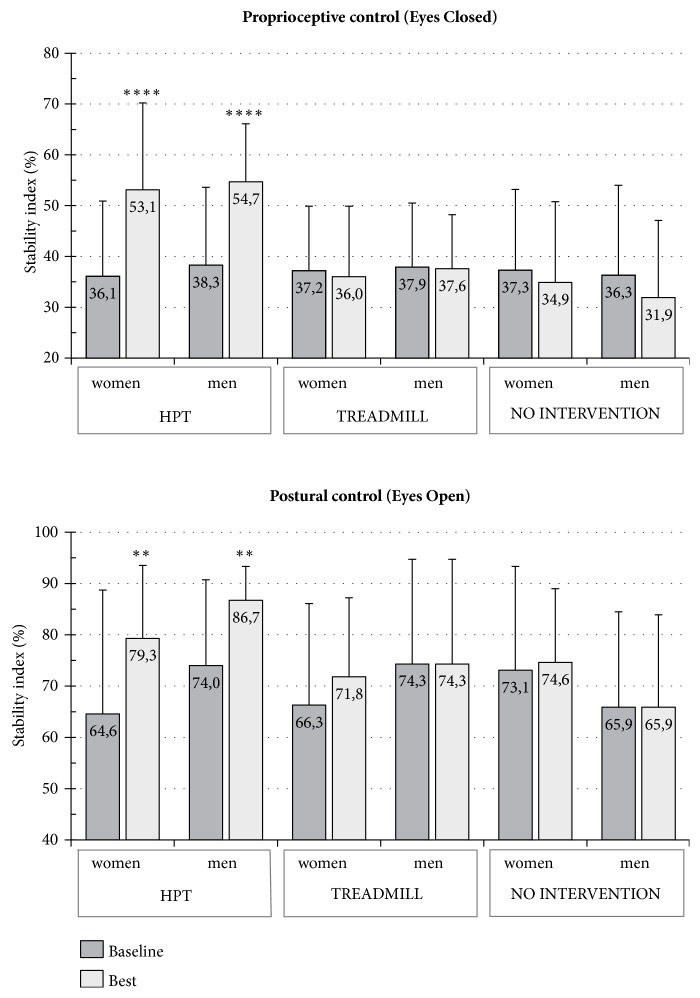
Static single stance test. Variations in proprioceptive control and postural control after 6 weeks of high-frequency proprioceptive training (HPT), treadmill training, or no intervention. Mean values ± SD; ^*∗∗∗∗*^p < 0.001, ^*∗∗*^p ≤ 0.01.

**Figure 3:**
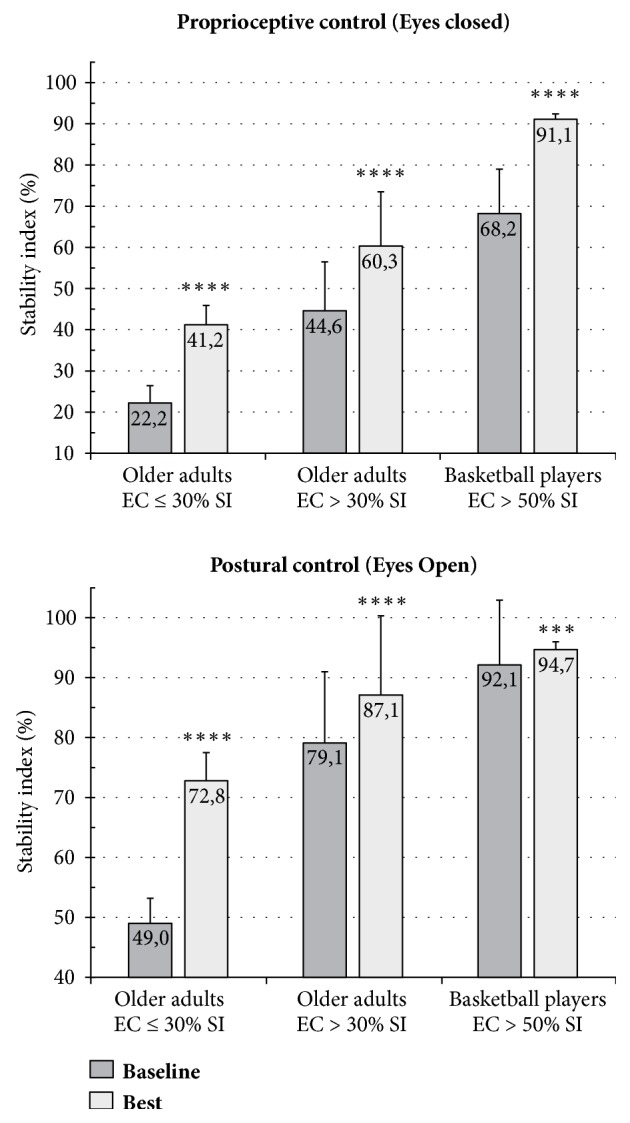
Static single stance test. Variations in proprioceptive control and postural control after 6 weeks of high-frequency proprioceptive training in three groups with different starting levels of proprioceptive control. EC = eyes closed. SI = stability index. Mean values ± SD; ^*∗∗∗∗*^p < 0.001, ^*∗∗∗*^p < 0.005.

**Figure 4:**
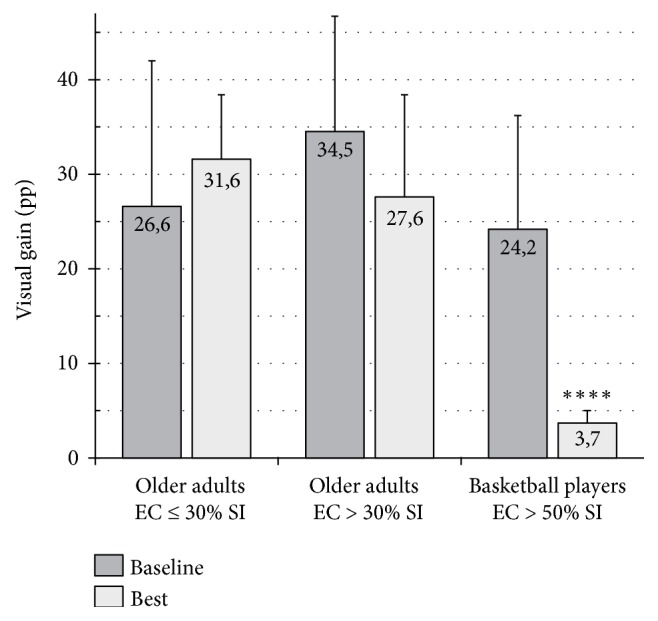
Variations in visual gain (static single stance test) after 6 weeks of high-frequency proprioceptive training in groups with different starting levels of proprioceptive control. EC = eyes closed (marker of proprioceptive control). SI = stability index. Mean values ± SD; ^*∗∗∗∗*^p < 0.001.

The characteristics of the 61 subjects in this study (30 males, 31 females) are summarized in Table 1. The three intervention groups did not significantly differ in baseline demographic characteristics and stability scores.  Table 4 and Figure 2 present the results of the six subgroups after 6 weeks of different training activities or no intervention. A comparison of the baseline test with the best test performed after the HPT demonstrated that both men and women had a significant increase in stability based on proprioceptive control (EC condition). The proprioceptive control improved by 17.0 pp in women (p<0.001, 95 % CI 9.53–24.54) and by 16.4 pp in men (p<0.001, 95 % CI 8.66–24.22). Postural control (stability in EO condition) increased by 14.7 in women, p<0.01, 95 % CI 6.01–23.28, and by 12.7 pp in men (p<0.01, 95 % CI 4.1–21.28). The response to HPT showed no gender difference but highlighted three different responses according to the proprioceptive level at baseline (Figures 3 and 4). As there were no differences between the genders, males and females were considered altogether. The subjects with a low-medium level of proprioception-based stability at baseline (SI in EC condition >30 %) presented a significant increase in proprioceptive control (15.7 pp, p<0.001, 95 % CI 8.12–23.20) and a moderate decrease in VG (-6.9 pp). Moreover, the subjects with extremely low proprioceptive control at baseline (SI in EC condition ≤30 %) frequently showed limited compensatory visual stabilization. After HPT, proprioceptive control (EC) significantly improved (18.9 pp, p<0.001, 95 % CI 16.20–21.66) and triggered an increase in VG (4.8 pp), thereby improving the stability in EO condition (23.8 pp, p<0.01, 95 % CI 11.60–35.87).

Twelve sessions of walking on a treadmill at increasing speed in 6 weeks failed to improve the proprioceptive control and postural control (EC and EO stability) in both genders. Similarly, the no-intervention group did not show any change in the parameters, in either men or women, compared to baseline values.

## 4. Discussion

The study investigated whether quantifiable HPT could improve single stance stability by increasing proprioceptive control and optimizing the contribution of vision as a compensatory stabilizer. The primary findings of this study are as follows.

1. HPT could significantly improve, in both genders, single stance stability based on proprioceptive control (EC stability). Improvement in proprioceptive control could also enhance postural control (EO stability) significantly, in most cases decreasing visual dependence or unblocking the stabilizing contribution of vision (see point (2)).
2. Different levels of proprioceptive control could activate different engagement of vision in response to proprioceptive training (Figures 3 and 4). The usual response to HPT consists of an increase in proprioceptive control and a reduction in the stabilizing contribution of vision. Our study showed that extremely low proprioceptive control (SI in EC condition ≤30 %) could limit VG as a compensatory stabilizer in EO condition; in this case, a moderate increase in proprioceptive control reactivates VG, which in turn improves postural control (EO stability) significantly. The visual system would require a minimum level of proprioceptive control (SI >30 % in EC condition) to activate the stabilizing contribution [17].
3. Women aged 75–84 years are significantly less stable than men [17]; this difference could be attributable to the more accentuated proprioceptive control impairment resulting from muscle weakness that reaches a critical level earlier in women than in men [24–26] and a more rapid proprioceptive disuse in women due to simplified interaction with the ground, including lifestyle [27], kind of shoes worn [28, 29], and decreased weight-bearing surface of the forefoot consequent to structural disorders (bunions, hammertoes, etc.) [17, 30]. Nevertheless, our study found that the males and females have similar response to HPT.
4. Walking on a treadmill at an increasing speed failed to significantly improve proprioceptive control and postural control (EC and EO stability, respectively), in both genders. A previous study [31] that investigated the effectiveness of forward and backward treadmill walking on postural stability among older people showed that stability in double stance but not in tandem stance improved. Single stance was not assessed. Pereira [32] investigated the long-term effects of walking on the health status. In this study, with a 10-year follow-up, no significant reduction in risk of falling was noted. Sherrington et al. [33] evaluated in a systematic review with meta-analysis of 44 randomized controlled trials examining the effects of physical activity on falls. Programs which included a walking program and only provided a low-to-moderate challenge to balance did not significantly reduce falls. According to Frank and Patla [34], older adults should walk on different terrains (e.g., uneven surfaces) and in differing environments to preserve the mobility independence and be prepared to face the environmental challenges of their daily living. Okubo et al. [35] found that brisk walking can be more effective than balance training in fall prevention among community-dwelling older adults. According to the author, the lack of consistency with most previous studies would depend on the enrollment of subjects with a high risk of falls. However, due to the absence of stability assessments, the real effect of the intervention programs on balance and its sensory components is unknown. Moreover, while the walking program was quantifiable by means of pedometers, balance and strength training were not quantifiable.

In developed countries, lack of interaction with uneven ground reduces reflex activation of the stabilizing muscles and minimizes the mechanical solicitations of the weight-bearing structures (e.g., ligaments, tendons, joints, bones of the lower limb, and spine). Thus, these structures become more fragile and the stabilizing muscles become weaker. Consequently, the single stance phase becomes more unstable mainly because of the weakness of the extrinsic and intrinsic muscles of the foot, which are unable to generate adequate forces to stabilize the weight-bearing leg and the upper part of the body. This is worsened by the structural frailty of the passive structures that are unable to serve as a strong anchorage point. Based on these findings, increasing the duration of walking on flat surfaces, even at a higher speed, is ineffective for increasing proprioceptive control (EC stability) and postural control (EO stability) in single stance.

### 4.1. Primary Role of Proprioception

The compensatory role of vision as a postural stabilizer makes the subject only apparently more stable. The necessity of maintaining the eyes anchored to the environment [36] could explain why stability depending on vision is limited and poorly adaptable. Hence, improving stance stability based on proprioceptive reflexes and with decreased visual dependence is crucial in preventing falls. This emphasizes the primary role of proprioceptive control as a vertical stabilizer [17, 37] and is confirmed by our results. Consequently, postural control heavily based on vision could lead to a dramatic loss of stability in case of visual impairment [17].

### 4.2. Proprioceptive Training

No specific definition of proprioceptive exercises acting on the unconscious component of proprioception exists [38]. Generally, the so-called proprioceptive exercises require the management of instability. However, management of instability is not enough to consider a proprioceptive training proposal effective [36]. In most proprioceptive training programs, identifying common and quantifiable biomechanical or physiological characteristics that could lead to specific adaptation is impossible. Moreover, frequency, duration, and intensity of training vary across studies and sometimes are not specified [39]. The main goal of effective proprioceptive training should be optimizing the reflexes of the stabilizing muscles closest to the support surface (particularly the intrinsic and extrinsic muscles of the foot), which could in turn minimize the counteractions of the upper parts of the body (trunk and arms) and the involvement of vision as a vertical stabilizer. In this study, the biomechanical and physiological characteristics of HPT were quantifiable and modifiable. The most important elements were the visual feedforward that shows real-time movements of the rocking base and the radius of instability of the rolling surface (Table 2). Their characteristics were critical for enhancing the frequency of instability of the rocking base.

### 4.3. Causal Chain Model

**Figure 5:**
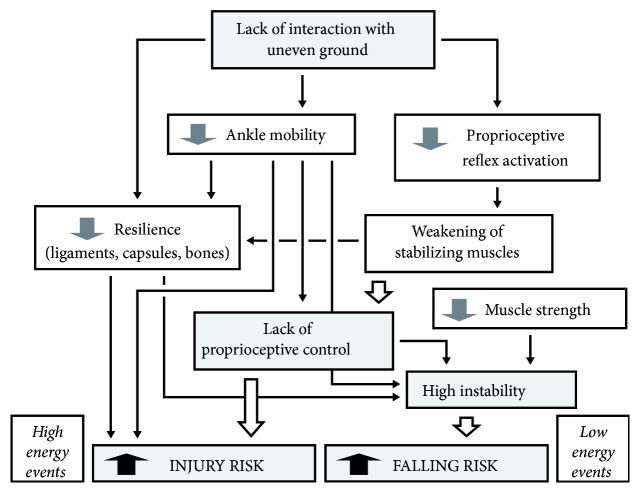
The causal chains that lead to an increasing intrinsic risk of falls in older adults and recurrent injuries in athletes. The initial determinant is the same. Injuries (like sprains) generally require events with high kinetic energy to occur and for this reason are unlikely in older adults that tend to move slower to counteract instability.

We speculated that lack of interaction with uneven ground is the initial determinant in the causal chain leading to lack of proprioceptive control and consequent high risk of falls in older adults. It is interesting to note that the results of the present study are consistent with those found in a previous six-year prospective study concerning injury prevention in a professional basketball team [21]. In that study, the athletes showed an inadequate level of proprioceptive control (SI range 50-70 % in EC condition) that HPT significantly improved to over 90 % (Figure 3), minimizing VG (Figure 4), with a dramatic reduction in the occurrence of ankle sprains and low back pain. On these bases, we hypothesized that lack of interaction with uneven ground and consequent lack of proprioceptive control could be the initial determinant even in the causal chain leading to recurrent injuries in athletes (Figure 5). These causal chains would be associated with the deactivation of proprioceptive reflexes and consequent weakening of the stabilizing muscles (above all the shank muscles), reduced mobility of the ankle joints, and decreased resilience of ligaments, capsules, and bones. These regressive adaptations were observed in both older adults and athletes because they always move on flat surfaces (floors, pavements, practice courts). Moreover, the study on injury prevention in basketball highlighted that top physical training, including classic proprioceptive exercises, was not able to guarantee a level of proprioceptive control which ensures high protection against injuries. Even if older adults and basketball players were different in terms of age, anthropometric characteristics, and performances [21], their responses to HPT were similar. An important difference between the two groups was that older adults need a training period 2.5–3 times longer than that of the athletes to generate the same number of mechanical solicitations (Table 3).

### 4.4. Limitations

The main limitation of this study could be the self-selection bias. People who volunteer to participate in a study tend to be different from the rest of the population (the former being more health conscious and better educated), thereby limiting the external validity of the results [40]. Nevertheless, the random assignment of eligible subjects willing to participate to the intervention groups should have minimized the selection bias.

Another limitation was the sample size. Analysis involving several age classifications and more functional levels is possible with a greater number of subjects.

## 5. Conclusions

HPT (6 weeks, 12 sessions) significantly improved single stance stability in older adults by increasing proprioceptive control and optimizing the contribution of vision as stabilizer. Different levels of proprioceptive control could minimize, inhibit, or activate the involvement of vision as a stabilizer. In cases with progressively lower values of proprioceptive control (SI in EC between 90 % and 30 %), the visual stabilizer tends to increase its compensatory action; in these cases, HPT results in increased proprioceptive control and reduced visual dependence to achieve stability. However, extremely low proprioceptive control (SI in EC ≤30 %) inhibits the compensatory role of vision as a stabilizer; in this case, a moderate increase in proprioceptive control (stability in EC condition) significantly activates VG, improving stability in the EO condition.

HPT would emerge as a powerful activator of refined proprioceptive control, which allows increased single stance stability, safer interaction with the ground, and mitigation of other risk factors. The effectiveness of HPT in strengthening the stabilizing muscles is also endorsed by the finding that treadmill walking, i.e., walking on a flat and smooth surface, does not significantly improve proprioceptive control and postural control (stability in EC and EO conditions, respectively), in both genders.

Older adults should start HPT before the risk of fall becomes evident to maximize the results of HPT (they need an HPT period 2.5-3 times longer than that for athletes to generate the same number of mechanical solicitations). Early HPT allows more intense succeeding HPT sessions with cumulative effects. Our preliminary results with a 10-year follow-up showed that 12–18 consecutive sessions per year (45 min, in 6–9 weeks) starting between the age of 60 and 75 years could determine a progressive and continuous improvement in stability. As instability takes more than ten years to become evident, an early preventive intervention would increase the results and reduce the cost. Considering that proprioceptive control in single stance could be an important element of safety and mobility independence, further research with long-term follow-ups on the cumulative effects of periodical HPT is suggested.

## Figures and Tables

**Table 1:** Subject characteristics.

Characteristics	HPT groups		Treadmill groups		No intervention groups	
	Women	Men	Women	Men	Women	Men
N. of subjects	11	10	10	10	10	10
Age (years)	70,6 ± 5,2	72,2 ± 5,0	72,9 ± 5,3	75,5 ± 4,4	74,0 ± 5,1	76,5 ± 5,3
Height (cm)	161,2 ± 4,0	173,4 ± 7,3	158,2 ± 7,1	173,5 ±3,0	159,1 ± 5,5	168,6 ± 7,2
Body weight (kg)	64,6 ± 10,1	72.0 ± 6,1	60,8 ± 16,4	77,4 ± 13,0	62,4 ± 8,3	69,2 ± 10,8
Body Mass Index (kg/m^2^)	24,9 ± 4,0	24,0 ± 2,1	24,3 ± 6,7	25,8 ± 4,7	24,7 ± 3,5	24,3 ± 3,1

Values are mean ± SD. HPT = high-frequency proprioceptive training.

**Table 2:** Characteristics of the rocking board.

Board Characteristics			
Typology of instability			Rocking
Rolling structure			Section of a cylinder
Radius of the rolling structure (changeable)			55-80-110 mm
Distance of the plantar surface from the ground			50 mm
Degrees of freedom			1 (single axis)

Range of motion	Mobile roll axis (x)	Inclination	± 15°
		Rolling	≈30-45-60 mm (rolling radius 55-80-110 mm)
	Mobile pitch axis (y)	Inclination	0°
		Rolling	0°
	Yaw axis (z)	Rotation	0°

Frequency enhancer			Feedback/feedforward

The rocking motion is a complex movement that includes the rolling of a cylindrical surface and the consequent inclination of the moving plate.

**Table 3:** 6-weeks' high-frequency proprioceptive training characteristics of older adults and athletes.

HPT Characteristics	Unit	Older adults (present study)	Professional athletes (previous study)
Frequency of instability		High	Very high

Single session duration	min	45	10–30
Weekly session number		2	2-4
Weekly HPT*∗* time	min	90	50 ± 5
Inter-trial recovery time	s	15–20	10–5
Weekly actual HPT time	min	40	45 ± 5
Density^a^	%	Low (<50)	High (>85)

HPT time	h	9	5
Actual HPT time^b^	h	4	4
Rocking inversions per hour	n	8,000–10,000	20,000–30,000

*∗* HPT = high-frequency proprioceptive training

^a^ Density = actual HPT time/session duration

^b^ 6-week period

**Table 4:** Static single stance test.

Groups	Gender	Condition	SI baseline (%)	SI best (%)	Delta (pp)	p	95 % CI
HPT	Women	EO	64.6 ± 24.1	79.3 ± 14.2	14.7	<0.005	6.01–23.28
		EC	36.1 ± 14.8	53.1 ± 17 .1	17.0	<0.001	9.53–24.54
	Men	EO	74.0 ± 16.7	86.7 ± 6.6	12.7	<0.01	4.1–21.28
		EC	38.3 ± 15.3	54.7 ± 11.4	16.4	<0.001	8.66–24.22

Treadmill	Women	EO	66.3 ± 19.8	71.8 ± 15.4	5.5	0.103	-1.36–12.38
		EC	37.2 ± 12.7	36.0 ± 13.9	-1.2	0.517	-5.40–2.92
	Men	EO	74.3 ± 20.4	70.6 ± 19.1	-3.7	0.099	-8.28–0.86
		EC	37.9 ± 12.6	37.6 ± 10.6	-0.3	0.866	-4.05–4.47

No intervention	Women	EO	73.1 ± 20.2	74.6 ± 14.4	1.5	0.687	-6.64–9.64
		EC	37.3 ± 15.9	34.9 ± 15.9	-2.4	0.547	-10.98–6.22
	Men	EO	65.9 ± 18.6	67.8 ± 17.1	1.9	0.534	-4.74–8.54
		EC	36.3 ± 17.7	31.9 ± 15.2	-4.4	0.167	-11.12-2.24

Significant improvements in proprioceptive control (EC) and postural control (EO) after 6 weeks of high-frequency proprioceptive training.

SI = stability index; EO = eyes open (marker of postural control); EC = eyes closed (marker of proprioceptive control); pp = percentage point; CI = confidence interval; HPT = high-frequency proprioceptive training.

## Data Availability

The data used to support the findings of this study are available from the corresponding author upon request.

## Supplementary Materials

Supplementary Materials Figure SM. Study participant flow.
